# Dynamic and integrative approaches to understanding pathogen spillover

**DOI:** 10.1098/rstb.2019.0014

**Published:** 2019-08-12

**Authors:** Daniel J. Becker, Alex D. Washburne, Christina L. Faust, Juliet R. C. Pulliam, Erin A. Mordecai, James O. Lloyd-Smith, Raina K. Plowright

**Affiliations:** 1 Department of Microbiology and Immunology, Montana State University, Bozeman, MT 59717, USA; 2 Center for the Ecology of Infectious Diseases, University of Georgia, Athens, GA, USA; 3 Department of Biology, Indiana University, Bloomington, IN, USA; 4 Institute of Biodiversity Animal Health and Comparative Medicine, University of Glasgow, Glasgow, UK; 5 South African Centre for Epidemiological Modelling and Analysis (SACEMA), Stellenbosch University, Stellenbosch, South Africa; 6 Department of Biology, Stanford University, Stanford, CA, USA; 7 Department of Ecology and Evolutionary Biology, University of California, Los Angeles, Los Angeles, CA, USA; 8 Fogarty International Center, National Institutes of Health, Bethesda, MD, USA

**Keywords:** cross-species transmission, epidemics, pandemics, zoonotic, surveillance, interventions

## 1. Introduction

Pathogen spillover is the process by which a pathogen is transmitted from a reservoir host species to a recipient host species [1,2]. The term is sometimes used more broadly, particularly in public discourse, blending in elements of onward transmission in the novel host species or even pathogen adaptation to the novel host [3,4]. This theme issue focuses on pathogen spillover *sensu stricto*, except where explicitly noted. Many of the examples considered pertain to zoonotic spillover (i.e. from wildlife or domestic animals to humans), given recent epidemics (e.g. Ebola virus [5]) and pandemics (e.g. H1N1 influenza virus [6]); however, we emphasize the general methods and mechanisms involved in understanding spillover between any two species, such as those that threaten wildlife conservation (e.g. *Mycoplasma ovipneumoniae* from domestic sheep to bighorn sheep [7]) and the agricultural sector (e.g. *Brucella abortus* from elk to cattle [8]).

Spillover requires the spatial and temporal alignment of several hierarchical factors that must occur for a pathogen to be transmitted from a reservoir or source host to a recipient host of a different species [9]. These factors include reservoir host distribution and abundance, pathogen prevalence and shedding from reservoir hosts; pathogen survival in the environment or arthropod vector; recipient host contact with the infectious agent, reservoir host or arthropod vector; and susceptibility of the recipient host. Following spillover, another suite of factors determines whether a pathogen is transmitted within the recipient host population (e.g. [10]). Research on pathogen spillover is often focused on a single component of this process through the lens of a particular discipline. For example, the distribution of reservoir hosts is often studied through ecology, contact between reservoirs and humans is often studied via epidemiology or anthropology, and the pathogenesis of zoonoses in humans is often studied with medical microbiology and immunology. While each factor must be studied and quantified, spillover is the emergent property of these collective processes. Studying each factor in isolation fails to account for the hierarchical and often nonlinear dynamics of the spillover system [9]. Pathogen surveillance, epidemic preparedness and management interventions would all benefit from integrative approaches that consider multiple components of pathogen spillover [1].

This theme issue stemmed from a 2018 workshop on cross-species transmission of pathogens, where participants from interlinked fields including ecology, mathematical modelling, epidemiology, virology and immunology discussed how to better understand and predict pathogen spillover. Here, we bring together a diverse set of perspectives—including empirical research, theory and synthetic reviews—to highlight cutting-edge research and to provide a roadmap for quantifying and integrating host–pathogen dynamics at each step in the spillover process. Manuscripts are organized around three approaches. The first set of manuscripts focuses on integrating data streams to understand spillover dynamics and predict risk. The second set of manuscripts focuses on in-depth analysis of each of the factors affecting cross-species transmission: infection dynamics in reservoir hosts, pathogen survival in the environment, recipient host exposure, dose–response relationships and establishment of infection in recipient hosts. The final set of manuscripts focuses on applied perspectives, with an emphasis on surveillance and interventions. Here, we summarize these contributions to highlight key insights, methodologies and future directions to improve our understanding of pathogen spillover.

## 2. Integrating data streams to understand spillover dynamics

Because spillover is the outcome of multiple ecological, epidemiological and immunological factors aligning in space and time [9,10], predictive frameworks aim to integrate data pertinent to these factors to quantify the relative importance of these processes and to estimate risk. Cross *et al*. [12] review approaches for estimating spatio-temporal variation in spillover risk, focusing on the wildlife–livestock interface. The authors highlight the challenges inherent in either correlating observed spillover events with relevant covariates or integrating data on host density, distribution and pathogen prevalence using mechanistic models. They highlight that mechanistic approaches may be especially useful in systems where spillovers are infrequent, rarely observed or hard to differentiate from within-species transmission; however, linking datasets on different factors in the spillover pathway requires that such datasets be related to a common spatial and temporal resolution. The authors use case studies of brucellosis in the Greater Yellowstone Ecosystem [13] and of avian influenza virus in China and North America [14] to emphasize potential solutions to these challenges for estimating spillover risk.

Emphasizing that statistical modelling efforts may struggle to detect nonlinear and stochastic relationships inherent in pathogen spillover, Childs *et al*. [15] provide a strong test of theory governing how hierarchical barriers control cross-species transmission [9]. The authors focus their case study on yellow fever, a mosquito-borne viral disease of historical importance in South America that persists in the region largely in sylvatic cycles that occasionally spill over to infect humans [16,17]. Specifically, they use mechanistic models that incorporate spatial ecological and immunological data from Brazil across 16 years to predict yellow fever spillover in humans. The authors show that a mechanistic model of spillover risk, based on the ecology of mosquito vectors and non-human primate reservoirs, best predicts spillover events compared with models that also include human population size and immunity. This result arises because spillover occurs even in areas with low human population density and high vaccination coverage (e.g. parts of the Amazon), so population density and vaccination coverage tend to inflate the predicted risk in locations with low ecological suitability. This integrated approach also highlights a key research gap—cyclical dynamics of susceptible primate populations—that could further improve prediction. This work illustrates that mechanistically modelling the interactions among the environment, viruses, vectors, non-human primates and humans can predict rare and seemingly stochastic spillover events with high accuracy.

Washburne *et al.* [17] study the general statistical problems that can arise when aiming to forecast spillover risk. The authors highlight that any such statistical efforts will compile a dataset of explanatory variables expected to relate to pre-spillover processes (e.g., infection prevalence in reservoirs, human vaccination coverage) that are aligned with one of two response variables: the presence and absence of spillover or the number of spillover events at some spatial and temporal resolution (e.g., spatio-temporal counts of yellow fever spillovers [15]). The authors show how modelling cross-species transmission as a percolation process, in which pathogens move from infected reservoirs to recipient hosts along a graph representing various spillover pathways [18,19], reveals first principles for how such datasets will behave and how common statistical tools can produce misleading inferences and poor predictions. For example, percolation theory reveals an inherent nonlinearity in modelling spillover counts, in which statistical inferences are driven by the dominant reservoir sources of infection and the most limiting barriers to cross-species transmission; this nonlinearity can mask the influence of alternative reservoir species or barriers, both of which could be modified through interventions but whose sensitivity as a management tool will appear reduced under linear models. Percolation models provide a conceptual framework to connect statistical and mechanistic models with applications to limit risk by illuminating unexpected statistical principles governing pathogen spillover and the nonlinear impacts of management actions.

## 3. Individual factors affecting pathogen spillover

The theme issue's second section uses empirical research, theory and synthetic reviews to understand the processes operating at each stage of pathogen spillover, from infection dynamics within the reservoir hosts to susceptibility and establishment of infection in the recipient host. For the former, the distribution and intensity of infection in reservoir hosts over space and time is the first determinant of spillover risk [9]. Data on these spatio-temporal dynamics help elucidate how pathogens circulate in reservoir hosts and when and where to expect pathogen excretion to be greatest [21]. However, such field data can be expensive and difficult to collect, and researchers inevitably must tradeoff between the extent and intensity of spatial versus temporal sampling. Sampling is thus often opportunistic and fails to adequately describe spillover. Plowright *et al*. [22] review factors that influence spatial and temporal variation in infection in reservoirs and describe sampling designs that can increase the quality and quantity of this information. Although the standard prescription from sampling theory is to sample randomly in space and time [23], probabilistic sampling designs are rare in the study of wildlife disease, given logistical challenges and non-random distributions of hosts. The authors highlight how stratified random sampling designs or adaptive sampling designs can help capture spatio-temporal pulses of infection when researchers have little *a priori* data on concentrations of infection or spillover events in space and time. These sampling designs can be integrated into modelling approaches and used to better quantify pathogen shedding from reservoirs. Accordingly, Glennon *et al*. [24] present a case study for how to use mechanistic models to differentiate among transmission processes for henipaviruses in straw-coloured fruit bats (*Eidolon helvum*). Using this virulent zoonosis as a case study, the authors generalize standard frameworks common in epidemiological modelling [25]. Given that henipavirus infection dynamics in bats are poorly understood, the authors study all possible transitions among infection states in bats to produce 46 potential models. Using likelihood-based methods, they fit these models to longitudinal data from captive bats to show strong support for reinfection after virus clearance and cycles of recurrent latent infection: key areas for future empirical work. This inclusive approach to confronting epidemiological models with longitudinal data in poorly understood reservoir host systems holds promise for elucidating spatio-temporal risk of pathogen spillover.

Following pathogen shedding from reservoir hosts, spillover risk is influenced by the duration of pathogen survival and possible reproduction outside the host in the environment [9]. For pathogens such as avian influenza virus, persistence in the environment (e.g. ponds) can also facilitate viral reassortment when strains co-occur, promoting co-infections during environmental exposure [26,27]. Pepin *et al*. [28] review and discuss how genomics, experimental ecology and epidemiological modelling can be leveraged to understand viral reassortment in environmental reservoirs. Although no gold standard for capturing, isolating and identifying avian influenza virus diversity from the environment exists, environmental metagenomics and field-based viral diagnostics (e.g. field-based nucleic acid extraction, PCR and sequencing) hold promise for characterizing this context of viral reassortment [29,30]. The authors note how standardizing such field protocols and coupling these data streams with quantitative disease models and natural transmission studies should dramatically improve our understanding of viral co-occurrence and reassortment and thus, this additional process in the pathway to spillover.

Exposure of recipient hosts to pathogens (e.g. those persisting in the environment) can take a variety of forms; however, in a more general sense, exposure often occurs at elevated rates near boundaries between ecosystems [31]. Borremans *et al*. [32] review how ecosystem boundaries can promote spillover by applying ecological theory to understand landscape permeability across ecosystems. The authors highlight that the traits of hosts and pathogens are critical for determining effects of ecosystem boundaries on cross-species transmission. Properties of ecosystem boundaries can also promote or inhibit exposure; for example, edge effects can affect species composition, diversity and population size between ecosystems, as can features of landscape configuration such as patch size and perimeter-to-area ratio [33]. By considering the analogy between parasite flow and resource flow and by applying concepts from movement ecology, Borremans *et al*. [32] connect contact rates and spillover risk across ecosystem boundaries to generalize between pathogens and integrate into broader ecological theory.

Following the complex interactions between reservoir hosts, vectors, pathogens, the environment and recipient hosts, a crucial juncture in any potential spillover event is the point when a recipient host is challenged with a given dose of pathogen (through a particular route and sometimes over a particular duration) and a successful infection does or does not ensue [9]. Lunn *et al*. [34] describe how the dose–response relationship, which quantifies the probability of successful infection in the recipient host as a function of challenge dose, can act as a filter on the aforementioned upstream dynamics to shape pathogen spillover risk. The authors integrate recent developments in the dose–response literature, as well as re-analysing data from animal challenge experiments with Nipah virus and Middle East respiratory syndrome coronavirus [35,36], to highlight challenges and opportunities arising at the intersection of infectious disease ecology, microbial risk assessment and virology. Lunn *et al*. [34] call for closer interactions between these fields and for a new generation of pathogen transmission models that link dose–response data to epidemiological dynamics. Gostic *et al*. [37] next provide an example of the epidemiological insights such an approach can yield. They present a modelling analysis of dose–response experiments for *Leptospira interrogans*, a globally important bacterial zoonosis for which environmental exposure to soil or water contaminated by urine of infected reservoir hosts is the primary transmission route [38]. By conducting well-designed challenge experiments across a range of exposure routes, and then developing a mechanistic model to identify and quantify the key barriers to infection, Gostic *et al*. [37] show that intact skin is the crucial defence against leptospiral infection and that skin abrasions or wounds can increase recipient host infection risk by at least a million-fold. This close integration of experimental and modelling approaches isolates a potent and well-defined risk factor for infection with *Leptospira*, opening the door to targeted interventions to reduce spillover risk.

Once a pathogen has crossed these within-host barriers to replicate and disseminate in the recipient host, the outcome of infection may range from subclinical illness to death and from dead-end spillover to sustained onward transmission [9]. Bonneaud *et al*. [39] focus on the conditions favouring pathogen emergence, from the initial jump into the recipient host to adaption in the novel host environment [40]. The authors highlight that our current understanding of host shifts stems primarily from viral infections, limiting generalizations to other pathogen taxa, given substantial differences in ecology and life history [41]. They propose several non-mutually exclusive hypotheses to explain why novel bacterial pathogens may be less likely to specialize on their novel hosts and then test these with a mathematical model. The authors demonstrate that high levels of phenotypic plasticity, low rates of evolution and the ability to recombine should reduce propensity to specialize, suggesting that novel bacterial infections may be more likely to result in transient spillovers or increased host ranges than in host shifts.

Wasik *et al*. [42] in turn describe the within-host barriers that pathogens, and viruses in particular, must overcome to replicate and spread in new host populations to cause onward transmission. They present three well-documented examples of viruses that have crossed these barriers to cause epidemics or pandemics in the new host species: influenza A viruses [43], human immunodeficiency virus [44] and canine parvovirus [45]. The authors emphasize the role of integrated models that consider all the steps required to go from exposure to spillover to epidemic or pandemic. Guth *et al*. [46] expand upon these ideas through a comparative study of host and viral traits that predict virulence and the capacity for onward transmission in recipient hosts (i.e. humans). By expanding a previous global dataset of viral zoonoses [47], the authors show that increasing reservoir host phylogenetic distance from humans positively correlates with human mortality but negatively correlates with human-to-human transmissibility, suggesting that the virulence induced by reservoirs at high phylogenetic distance may limit viral capacity for onward transmission [48]. In particular, distantly related reservoirs, such as bats, harbour highly virulent zoonotic viruses with a lower capacity for onward transmission in recipient human hosts, building upon prior work describing bats as special reservoirs [49].

## 4. Applications for management of spillover

The theme issue's final section focuses on applied perspectives to detect early spillover events (i.e. surveillance) and the role of interventions focused upstream in the spillover pathway. In particular, early detection is critical for minimizing the spread of zoonotic pathogens following an initial spillover event [50]. A first series of manuscripts emphasize different approaches to the surveillance of zoonoses. Schmidt *et al*. [51] use machine learning tools (e.g. boosted regression trees [52]) to predict which mammal species are more likely to play roles in Ebola virus spillover events. The authors show that large-bodied, frugivorous mammals with slow life histories are likely host species, implicating some insectivorous bats, Old World monkeys and forest antelopes as possible Ebola virus reservoirs. Predictions such as these can help prioritize future wildlife surveillance efforts (e.g. [53]). Kuisma *et al*. [54] in turn describe a community-based surveillance effort focused on wildlife mortality reporting and oriented to early detection of Ebola virus disease outbreaks. Spanning over a decade and covering 50 000 km^2^ of challenging terrain in the Congo basin, this programme has reached hundreds of villages and thousands of hunters and forest gatherers. The programme has educated community members in wildlife carcass reporting and behavioral risk reduction as well as built capacity for safe carcass sampling by trained local responders. This region was not confronted with an Ebola virus outbreak during the period described here, and all reported carcasses tested negative. Nevertheless, given the well-recognized fact that early intervention can avert massive human and economic costs of widespread epidemics, the low-cost and scalable surveillance programme described by the authors could provide key early detection capability more generally.

Two other contributions focus on zoonotic pathogen surveillance efforts in domestic animals and human populations. Mwangi *et al*. [55] present a real-time surveillance system that leverages the existing mobile phone network and shows immense potential to improve adaptive management of spillover. This surveillance system has been implemented in 1500 households across rural Kenya, where participants are asked to report symptom syndromes in their livestock. Zoonotic diseases such as Rift Valley fever present with severe clinical signs in domestic animal populations, but lack of active surveillance can miss these sentinels [56]. The authors demonstrate that illnesses were more likely to be reported on mobile phones compared with standard routine household animal surveys. They also show that more severe symptoms are likely to be reported, highlighting the utility of this surveillance method for diseases such as Rift Valley fever. Das *et al*. [57] similarly describe the implementation of a surveillance system in hospitals in Bangladesh that screens symptomatic patients for potential zoonoses. Most patients did not have a laboratory diagnosis for their illness, indicating that unidentified pathogens are likely spilling over in human populations. Broad-scale, sustainable human surveillance programmes such as outlined by the authors can play a critical role in early detection of zoonotic spillovers.

Following these approaches to surveillance, interventions can accordingly focus upstream or downstream in the pathway to spillover, given available data and resources, to limit cross-species transmission. At the wildlife–livestock interface, managing pathogen spillover is a main goal for animal husbandry, conservation and food security [58]. Yet, managers are often forced to make control decisions on the basis of limited evidence about intervention efficacy. Manlove *et al*. [59] develop a spatially explicit, stochastic model of pathogen transmission within and between wildlife reservoirs and livestock recipient hosts to improve evidence-based decision-making. By varying host movement patterns and epidemic growth rates, the authors show that biosecurity, containment and retroactive vaccination of the reservoir are the most effective for limiting the spatial spread and magnitude of spillover risk for fast-moving epidemics in mobile hosts. By contrast, prophylactic vaccination and depopulation of the reservoir host were more successful for fast-moving epidemics with low rates of host movement. This framework provides general intuition for how to manage different pathogens at the wildlife–livestock interface, and a flexible platform for more rigorously investigating disease control strategies.

Ultimately, one of the primary goals of research focused on pathogen spillover is to design interventions that can reduce or eliminate disease burden in recipient hosts. Sokolow *et al*. [60] explore how ecological interventions, which target the ecological context in which cross-species transmission occurs, can complement more traditional biomedical and veterinary interventions (e.g. vaccination, culling). The authors provide case studies to illustrate the potential for ecological interventions that target the reservoir host (sometimes indirectly, such as through the restoration of natural enemy populations [61]), pathogen survival in the environment, contact between reservoir and recipient hosts, or other aspects of risk in the recipient species. The authors also present a simple mechanistic model, parameterized for two example systems, that shows how nonlinear effects can produce counterintuitive results when comparing potential intervention strategies and highlights the importance of a detailed understanding of underlying ecological dynamics when designing and assessing interventions. Lastly, the authors draw attention to the importance of social, economic and political considerations to intervention success, as these can derail even the most efficient or cost-effective intervention. In particular, aligning the benefits of an intervention with the costs incurred is crucial to motivate ecological interventions and may require working across sectors for successful implementation.

## 5. Future directions and conclusion

Pathogen spillover is the result of a complex series of events that result in the successful establishment of infection in a recipient host [9]. As highlighted in the final paper of this theme issue, developing actionable forecasts of risk is further complicated by the various phylogenetic, spatial and temporal scales over which we study and predict spillover [62]. The authors here contextualize a diverse range of approaches to pathogen spillover within these scales to illustrate critical areas of pragmatic overlap. By focusing on an ecological perspective, the authors outline a research pipeline that connects pathogen discovery and macroecological analyses with spatio-temporal surveillance in reservoir and recipient hosts. Through several case studies (e.g. Lyme disease [63], Hendra virus [64], *Plasmodium knowlesi* [65]), the authors further demonstrate how ecologically focused research has facilitated predicting spillover of particular pathogens in space and time and facilitated design of intervention strategies. This synthesis shows how greater integration of macroecology, pathogen discovery and surveillance could ultimately generate more actionable predictions and interventions to limit spillover risks.

Recent epidemics, pandemics and disease emergence events all underscore the need to improve approaches to predict and prevent pathogen spillover. This theme issue highlights a range of methods and their commonalities through diverse host–pathogen systems for which researchers are assessing factors driving spillover risk across varying phylogenetic, spatial and temporal scales. Contributing manuscripts further emphasize how developing a mechanistic understanding of the hierarchical factors affecting spillover can facilitate quantifying the drivers of cross-species transmission, deriving generalizable theory and making robust predictions, even for seemingly rare and idiosyncratic spillover events. Importantly, such insights can improve our ability to deploy surveillance efforts, design interventions at early stages of the pathway to spillover and manage disease cases in recipient hosts, thereby limiting or preventing further outbreaks. Continued study of pathogen spillover as a repeated and hierarchical phenomenon will only improve our ability to predict, prevent and manage cross-species transmission risks.

## Data accessibility

This manuscript has no additional data.
